# The layered polyphosphide Ag_3.73(4)_Zn_2.27(4)_P_16_

**DOI:** 10.1107/S1600536812045667

**Published:** 2012-11-10

**Authors:** Marianne Köpf, Oliver Osters, Melanie Bawohl, Tom Nilges

**Affiliations:** a Technische Universität München, Department Chemie, Lichtenbergstrasse 4, 85747 Garching bei München, Germany

## Abstract

The silver zinc hexa­deca­phosphide Ag_3.73(4)_Zn_2.27(4)_P_16_ is the first polyphosphide in the ternary system Ag/Zn/P. It was synthesized from stoichiometric mixtures of Ag, Zn and P in the molar ratio 4:2:16, using AgI as a mineralizing agent in a gas-phase-assisted reaction. Ag_3.73(4)_Zn_2.27(4)_P_16_ crystallizes in the Cu_5_InP_16_ structure type. The asymmetric unit contains two Ag/Zn sites with mixed occupancies and four P sites. One of the Ag/Zn sites is located on a twofold rotation axis. The polyanionic [P_16_]-substructure consists of corrugated six-membered rings that are connected into a layer *via* the 1-, 2-, 4- and 5-positions of the rings by a bridging P atom in each case. The layers extend parallel to the *bc* plane and are stacked along the *a* axis. Both Ag/Zn sites are tetra­hedrally coordinated by P atoms.

## Related literature

For background to and structures of related polyphosphides, see: Bawohl & Nilges (2009 ▶); Dommann *et al.* (1989 ▶); Edmunds & Qurashi (1951 ▶); Lange *et al.* (2008 ▶); Möller & Jeitschko (1981 ▶); Olofsson (1965 ▶); Zanin *et al.* (2003 ▶). For background to the extinction correction, see: Becker & Coppens (1974 ▶).

## Experimental

### 

#### Crystal data

Ag_3.73_Zn_2.27_P_16_
*M* *_r_* = 1046.3
Monoclinic, 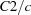

*a* = 11.492 (1) Å
*b* = 9.9604 (8) Å
*c* = 7.7106 (9) Å
β = 109.585 (9)°
*V* = 831.5 (2) Å^3^
*Z* = 2
Mo *K*α radiation
μ = 9.05 mm^−1^
*T* = 293 K
0.02 × 0.02 × 0.02 mm

#### Data collection

IPDS Stoe 2T diffractometer
Absorption correction: numerical (*X-AREA*; Stoe & Cie, 2011 ▶) *T*_min_ = 0.730, *T*_max_ = 0.771
4398 measured reflections
1265 independent reflections
1135 reflections with *I* > 3σ(*I*)
*R*_int_ = 0.015

#### Refinement

*R*[*F* ^2^ > 2σ(*F* ^2^)] = 0.018
*wR*(*F* ^2^) = 0.042
*S* = 1.39
1265 reflections
54 parameters
Δρ_max_ = 0.56 e Å^−3^
Δρ_min_ = −0.66 e Å^−3^

### 

Data collection: *X-AREA* (Stoe & Cie, 2011 ▶); cell refinement: *X-AREA*; data reduction: *X-AREA*; program(s) used to solve structure: *SUPERFLIP* (Palatinus & Chapuis, 2007 ▶); program(s) used to refine structure: *JANA2006* (Petřiček *et al.*, 2006 ▶); molecular graphics: *DIAMOND* (Brandenburg & Putz, 2005 ▶); software used to prepare material for publication: *publCIF* (Westrip, 2010 ▶).

## Supplementary Material

Crystal structure: contains datablock(s) global, I. DOI: 10.1107/S1600536812045667/wm2694sup1.cif

Structure factors: contains datablock(s) I. DOI: 10.1107/S1600536812045667/wm2694Isup2.hkl

Additional supplementary materials:  crystallographic information; 3D view; checkCIF report

## Figures and Tables

**Fig. 1.**
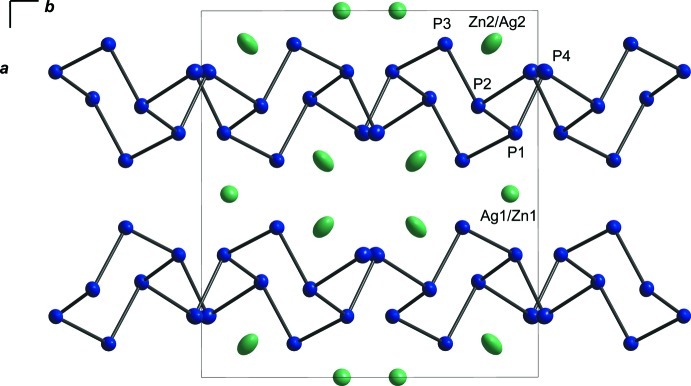
The crystal structure of Ag3.73 (4)Zn2.27 (4)P16, viewed along the c axis. The mixed Ag/Zn sites are drawn in turqueous and P atoms in blue, respectively. The displacement ellipsoids are shown at the 90% probability level.

## supplementary crystallographic information

### Comment

Herein we report on Ag_3.73 (4)_Zn_2.27 (4)_P_16_ (silver zinc hexadecaphosphide), the first ternary compound in the system Ag/Zn/P. It crystallizes isostructurally to Cu_5_InP_16_ in space group *C*2/*c* (Lange *et al.*, 2008). The asymmetric unit is built up by two mixed-occupied Ag/Zn sites and four P sites. Both Ag/Zn sites are tetrahedrally coordinated by four P atoms featuring bond lengths of 2.4386 (7) to 2.5432 (7) Å. The bond lengths of the (Ag1/Zn1) site to phosphorus range from 2.4386 (7) to 2.4836 (7) Å while slightly longer bond lengths of 2.4551 (7) to 2.5432 (7) Å are observed for (Ag2/Zn2). This finding is consistent with the higher amount of zinc on the (Ag1/Zn1) site leading to somewhat shorter bond lengths. For comparison, Zn—P bond length range from 2.36 to 2.40 Å in ZnP_2_ (Zanin *et al.*, 2003) and ZnP_4_ (Dommann *et al.*, 1989) while common Ag—P distances in polyphosphides are observed from 2.47 to 2.61 Å in Ag_3_P_11_ (Möller & Jeitschko, 1981) and 2.50 to 2.69 Å in AgP_2_ (Olofsson, 1965), respectively. Mixed-occupied Ag/Zn sites are not uncommon and are observed, for example, in intermetallic phases like Ag_4.5_Zn_4.5_ (or better AgZn; Edmunds & Qurashi, 1951). All P—P distances in Ag_3.73 (4)_Zn_2.27 (4)_P_16_, ranging from 2.1909 (9) to 2.2328 (9) Å, are within the expected range for polyphosphides (Bawohl & Nilges, 2009).

### Experimental

Ag_3.73 (4)_Zn_2.27 (4)_P_16_ was prepared by reaction from the elements Ag (ChemPur, powder, 99.9%), Zn (Sigma-Aldrich, pices, 99.9%), and P (ChemPur, powder, 99.999%) in the stoichiometric ratio of 4:2:16 in a 500 mg batch. As mineralizing agent, 10 mg AgI (ChemPur, powder, 99.9%) per 500 mg total sample weight was added. The reaction was carried out in evacuated ampoules in a muffle furnace at 823 K during 14 days using a heating ratio of 70 K/h. The sample was cooled down slowly at a rate of 5 K/h. For X-ray powder phase analyses a fraction of the sample was ground. Phase purity has been substantiated. Single crystals of suitable size for a single-crystal structure determination could be separated from the bulk phase. The sample was stable under atmospheric conditions for months.

### Refinement

The mixed-occupied Ag/Zn sites are located on Wyckoff positions 4*e* and 8*f*. The refinement of the Ag and Zn content was constrained to an overall full occupancy according to the sum of the two elements, each of them located on the same coordinates and with the same displacement parameters. The ratio of the two elements has been refined unrestricted.

### Crystal data

**Table:**

Ag_3.73_Zn_2.27_P_16_	*F*(000) = 966
*M**_r_* = 1046.3	*D*_x_ = 4.17 Mg m^−^^3^
Monoclinic, *C*2/*c*	Mo *K*α radiation, λ = 0.71073 Å
Hall symbol: -C 2yc	Cell parameters from 4528 reflections
*a* = 11.492 (1) Å	θ = 2.8–30.5°
*b* = 9.9604 (8) Å	µ = 9.05 mm^−^^1^
*c* = 7.7106 (9) Å	*T* = 293 K
β = 109.585 (9)°	Isomorphic, black
*V* = 831.5 (2) Å^3^	0.02 × 0.02 × 0.02 mm
*Z* = 2	

### Data collection

**Table:**

IPDS Stoe 2T diffractometer	1265 independent reflections
Radiation source: X-ray tube	1135 reflections with *I* > 3σ(*I*)
Plane graphite monochromator	*R*_int_ = 0.015
Detector resolution: 6.67 pixels mm^-1^	θ_max_ = 30.5°, θ_min_ = 2.8°
rotation method scans	*h* = −16→16
Absorption correction: numerical (*X-AREA*; Stoe & Cie, 2011)	*k* = −13→14
*T*_min_ = 0.730, *T*_max_ = 0.771	*l* = −10→10
4398 measured reflections	

### Refinement

**Table:**

Refinement on *F*^2^	14 constraints
*R*[*F*^2^ > 2σ(*F*^2^)] = 0.018	Weighting scheme based on measured s.u.'s *w* = 1/(σ^2^(*I*) + 0.0004*I*^2^)
*wR*(*F*^2^) = 0.042	(Δ/σ)_max_ = 0.038
*S* = 1.39	Δρ_max_ = 0.56 e Å^−^^3^
1265 reflections	Δρ_min_ = −0.66 e Å^−^^3^
54 parameters	Extinction correction: B-C type 1 Gaussian isotropic (Becker & Coppens, 1974)
0 restraints	Extinction coefficient: 0.084 (5)

### Fractional atomic coordinates and isotropic or equivalent isotropic displacement parameters (Å^2^)

**Table:**

	*x*	*y*	*z*	*U*_iso_*/*U*_eq_	Occ. (<1)
Ag1	0	0.41699 (3)	0.25	0.01075 (10)	0.422 (6)
Zn1	0	0.41699 (3)	0.25	0.01075 (10)	0.578 (6)
Ag2	−0.089927 (17)	0.13768 (2)	−0.08750 (3)	0.01608 (7)	0.721 (7)
Zn2	−0.089927 (17)	0.13768 (2)	−0.08750 (3)	0.01608 (7)	0.279 (7)
P1	−0.16624 (5)	0.56752 (5)	0.05996 (7)	0.00731 (15)	
P2	−0.24029 (5)	0.32264 (5)	−0.24701 (7)	0.00785 (15)	
P3	0.09157 (4)	0.27552 (6)	0.07194 (7)	0.00827 (15)	
P4	−0.33325 (5)	0.48032 (5)	−0.14361 (8)	0.00915 (15)	

### Atomic displacement parameters (Å^2^)

**Table:**

	*U*^11^	*U*^22^	*U*^33^	*U*^12^	*U*^13^	*U*^23^
Ag1	0.01067 (15)	0.01146 (16)	0.01117 (17)	0	0.00506 (10)	0
Zn1	0.01067 (15)	0.01146 (16)	0.01117 (17)	0	0.00506 (10)	0
Ag2	0.01637 (11)	0.01572 (12)	0.01331 (12)	−0.00637 (6)	0.00121 (7)	−0.00034 (6)
Zn2	0.01637 (11)	0.01572 (12)	0.01331 (12)	−0.00637 (6)	0.00121 (7)	−0.00034 (6)
P1	0.0079 (2)	0.0074 (2)	0.0071 (2)	0.00021 (16)	0.00310 (17)	0.00069 (17)
P2	0.0090 (2)	0.0076 (2)	0.0072 (2)	−0.00064 (16)	0.00309 (17)	−0.00022 (18)
P3	0.0077 (2)	0.0082 (2)	0.0093 (3)	0.00004 (16)	0.00342 (18)	0.00121 (17)
P4	0.0096 (2)	0.0104 (2)	0.0080 (2)	−0.00069 (17)	0.00363 (17)	−0.00171 (18)

### Geometric parameters (Å, º)

**Table:**

Ag1—P1	2.4836 (7)	Ag2—P4^iii^	2.5280 (7)
Ag1—P1^i^	2.4836 (7)	P1—P2^iv^	2.2328 (9)
Ag1—P3	2.4385 (7)	P1—P3^v^	2.1909 (9)
Ag1—P3^i^	2.4385 (7)	P1—P4	2.2095 (9)
Ag2—P2	2.5432 (7)	P2—P3^vi^	2.1976 (8)
Ag2—P3	2.4551 (7)	P2—P4	2.1941 (9)
Ag2—P4^ii^	2.5115 (7)		
			
P1—Ag1—P1^i^	105.73 (2)	Ag2—P2—P4	132.64 (3)
P1—Ag1—P3	114.11 (2)	P1^vii^—P2—P3^vi^	104.18 (3)
P1—Ag1—P3^i^	106.81 (2)	P1^vii^—P2—P4	103.43 (3)
P1^i^—Ag1—P3	106.81 (2)	P3^vi^—P2—P4	96.77 (3)
P1^i^—Ag1—P3^i^	114.11 (2)	Ag1—P3—Ag2	98.66 (2)
P3—Ag1—P3^i^	109.40 (2)	Ag1—P3—Zn2	98.66 (2)
P2—Ag2—P3	99.36 (2)	Ag1—P3—P1^v^	99.15 (3)
P2—Ag2—P4^ii^	93.38 (2)	Ag1—P3—P2^viii^	110.66 (3)
P2—Ag2—P4^iii^	109.66 (2)	Ag2—P3—P1^v^	124.46 (3)
P3—Ag2—P4^ii^	140.08 (2)	Ag2—P3—P2^viii^	119.19 (3)
P3—Ag2—P4^iii^	110.23 (2)	P1^v^—P3—P2^viii^	102.39 (3)
P4^ii^—Ag2—P4^iii^	100.52 (2)	Ag2^ix^—P4—Ag2^iii^	139.72 (3)
Ag1—P1—P2^iv^	106.99 (3)	Ag2^ix^—P4—Zn2^iii^	139.72 (3)
Ag1—P1—P3^v^	111.18 (3)	Ag2^ix^—P4—P1	108.81 (3)
Ag1—P1—P4	119.67 (3)	Ag2^ix^—P4—P2	103.06 (3)
P2^iv^—P1—P3^v^	104.82 (3)	Ag2^iii^—P4—Zn2^ix^	139.72 (3)
P2^iv^—P1—P4	103.40 (3)	Ag2^iii^—P4—P1	96.17 (3)
P3^v^—P1—P4	109.44 (3)	Ag2^iii^—P4—P2	104.53 (3)
Ag2—P2—P1^vii^	109.17 (3)	P1—P4—P2	97.28 (3)
Ag2—P2—P3^vi^	107.16 (3)		

Symmetry codes: (i) −*x*, *y*, −*z*+1/2; (ii) −*x*−1/2, *y*−1/2, −*z*−1/2; (iii) −*x*−1/2, −*y*+1/2, −*z*; (iv) *x*, −*y*+1, *z*+1/2; (v) −*x*, −*y*+1, −*z*; (vi) *x*−1/2, −*y*+1/2, *z*−1/2; (vii) *x*, −*y*+1, *z*−1/2; (viii) *x*+1/2, −*y*+1/2, *z*+1/2; (ix) −*x*−1/2, *y*+1/2, −*z*−1/2.
